# Treatment of Refractory Lactic Acidosis With Thiamine Administration in a Non-alcoholic Patient

**DOI:** 10.7759/cureus.16267

**Published:** 2021-07-08

**Authors:** Vihitha Thota, Mahati Paravathaneni, Sudheer Konduru, Bhanusowymya C Buragamadagu, Manaswitha Thota, Gabriel Lerman

**Affiliations:** 1 Internal Medicine, Mercy Catholic Medical Center, Darby, USA; 2 Internal Medicine, Virginia Commonwealth University School of Medicine, Richmond, USA; 3 Critical Care, Mercy Catholic Medical Center, Darby, USA

**Keywords:** lactic acid, lactic acidosis, thiamine, anaerobic metabolism, sepsis

## Abstract

Lactate, or lactic acid, is an end-product of anaerobic metabolism. The build-up of lactate in the body is commonly due to type A lactic acidosis, resulting from an inability to meet the body’s oxygen delivery demands. When lactic acidosis persists, other causes need to be ruled out. Here, we describe the case of a 63-year-old female who initially presented with hypoglycemia and was found to have significant lactic acidosis. Her blood sugar levels improved with dextrose supplementation; however, lactic acidosis persisted despite fluid hydration and empiric antibiotics. After excluding other causes of lactic acidosis, she was started on intravenous thiamine due to suspicion of thiamine deficiency secondary to poor nutrition. Lactic acid levels improved drastically after starting thiamine supplementation. Thiamine is a water-soluble vitamin that plays an essential role as a cofactor in several biochemical reactions. Thiamine deficiency is a rare, underdiagnosed cause of type B lactic acidosis, with early diagnosis and intervention playing crucial roles in preventing severe cardiac and neurological impairment.

## Introduction

Lactate is used in the critical care setting as a prognostic indicator, with lactic acidosis often associated with high mortality and adverse outcomes [1]. There are many causes of lactic acidosis, including hypoxemia, hypoperfusion, medications, and sepsis. A rare and under-recognized cause of type B lactic acidosis is severe thiamine deficiency, often seen in chronic alcoholism, gastrointestinal (GI) malabsorption, and in underdeveloped countries secondary to poor nutrition [2]. We present the case of a patient without a history of alcoholism or bowel surgery who was found to have severe refractory lactic acidosis secondary to thiamine deficiency.

## Case presentation

A 63-year-old female with a past medical history of congestive heart failure (CHF) with preserved ejection fraction, cerebral vascular accident with residual right-sided weakness, paroxysmal atrial fibrillation on apixaban, hypertension, and type 2 diabetes mellitus on insulin therapy presented to our hospital from a nursing home after persistent nausea and vomiting. Prior to arrival, as she was found to be lethargic, with a capillary glucose level of 21 mg/dL, she was given juice and dextrose in the nursing home; however, she was persistently hypoglycemic. Emergency Medical Services was called and she was started on intravenous (IV) D10 (10% dextrose in water) infusion, with blood glucose of 86 mg/dL upon arrival to the hospital. Upon presentation, her vital signs were notable for tachycardia with a heart rate of 99 beats per minute and low blood pressure of 104/55 mmHg (mean arterial pressure [MAP] of 71 mmHg). Her physical examination was notable for lethargy and positive stool hemoccult test. Initial bloodwork revealed mild leukocytosis, normocytic anemia, elevated anion gap metabolic acidosis, acute kidney injury with a baseline creatinine of 1.1 mg/dL, and elevated liver function tests (Table 1). Venous blood gas revealed a pH of 7.25, pCO_2_ 27.3 mmHg, bicarbonate (HCO_3_) 12 mmol/L, and lactate of >24 mmol/L (Table 2). She was admitted to the intensive care unit (ICU) for GI hemorrhage and severe lactic acidosis with concern for septic shock.

**Table 1:** Initial blood work done on admission. WBC: white blood cells; RBC: red blood cells; Hgb: hemoglobin; Hct: hematocrit; MCV: mean corpuscular volume; BUN: blood urea nitrogen; GFR: glomerular filtration rate; AST: aspartate aminotransferase; ALT: alanine aminotransferase; H: high; L: low

	Initial blood work on admission
WBC (4,500-11,000/µL)	12.5 H
RBC (4.2-5.4 × 10^6^/µL)	2.68 L
Hgb (12-16 g/dL)	4.7 L
Hct (36-46%)	16.9 L
MCV (80-100 fL)	85.7
Platelets (150,000-450,000/µL)	397
Sodium (136-145 mEq/L)	137
Potassium (3.5-5 mEq/L)	3.2 L
Chloride (98-110 mmol/L)	87 L
Carbon dioxide (23-29 mmol/L)	12 L
Anion gap (6-15)	38 H
BUN (7-25 mg/dL)	48 H
Creatinine (0.6-1.3 mg/dL)	1.6 H
Estimated GFR (>60 mL/min)	33
Glucose (70-110 mg/dL)	81
Total bilirubin (0.2-1.2 mg/dL)	1.4 H
Direct bilirubin (0-0.2 mg/dL)	0.8 H
AST (10-40 U/L)	43 H
ALT (7-52 U/L)	16
Alkaline phosphatase (35-110 U/L)	213 H
Albumin (3.5-5 g/dL)	2.6 L

**Table 2:** Blood gases throughout the patient’s hospital stay with a trend of acidosis and lactate levels. VBG: venous blood gas; ABG: arterial blood gas; H: high; L: low

	Day 1 1000 VBG	Day 1 1300 VBG	Day 1 1440 ABG	Day 2 0900 VBG
pH	7.25 L	7.15 L	7.31 L	7.44
pCO_2_ (mmHg)	27.3 L	29.3 L	22.5 L	51.2 H
pO_2_ (mmHg)	29.5 L	57.7 H	144 H	24.9 L
HCO_3_ (mmol/L)	12.0 L	10.3 L	11.4 L	34.4 H
Base excess (mmol/L)	-14.0 L	-16.8 L	-13.7 L	9.4 H
Sodium (mmol/L)	137	137	135	137
Potassium (mmol/L)	3.0 L	2.9 L	2.9 L	2.9 L
Chloride (mmol/L)	89 L	90 L	90 L	89 L
Lactate (0.4-2 mmol/L)	>24.0 H	>24.0 H	>24.0 H	5.2 H
Temperature (37ºC)	37	37	37	37

Apixaban was held, and the patient received three units of packed red blood cells for acute blood loss anemia, with improvement in hemoglobin to 7.8 g/dL. For lactic acidosis and suspected hypovolemia from blood loss, she received appropriate fluid resuscitation before being started on normal saline maintenance fluids, along with close monitoring of her respiratory and volume status in the setting of well-compensated CHF. Given the extent of the patient’s lactic acidosis and symptoms, she was started on broad-spectrum antibiotics with IV vancomycin and piperacillin/tazobactam. Bowel ischemia was also ruled out, with the patient having a benign abdominal examination and unremarkable computed tomography scan of the abdomen. Abdominal ultrasound showed no evidence of liver disease. Despite antibiotics and fluid resuscitation, serial blood gases persistently revealed lactate of >24 mmol/L (Table 2), with worsening acidemia. Serum lactic acid level was also elevated at 24.4 mmol/L. After 24 hours of hospitalization, the patient’s mentation significantly improved with the correction of hypoglycemia, and a more detailed history was obtained. The patient confirmed she had not been on metformin for many years prior to presentation and had been dependent on insulin. In addition, she revealed that she had not eaten for several weeks as she disliked the food at her nursing home and endorsed several weeks of upper extremity weakness. At this point in her clinical course, she had been appropriately volume resuscitated with correction of her creatinine to a baseline of 1.1 mg/dL and improvement in blood pressure to systolic blood pressure of 120-130 mmHg over diastolic blood pressure of 70 mmHg (MAPs 80-90 mmHg). Hemoglobin also continued to improve, with colonoscopy revealing a resolved diverticular bleed. Despite improvement in clinical status, with hemoglobin of 9.1 g/dL, lactic acid remained elevated at >24 mmol/L (Table 2).

Thorough medication reconciliation was also done; however, the patient was not on any agents that could cause lactic acidosis. Therefore, after ruling out other etiologies of lactic acidosis, thiamine deficiency was suspected clinically secondary to poor nutritional status. Thiamine was administered empirically before testing due to high clinical suspicion and anticipated delay in obtaining results due to the test being a send-out test in our hospital. She was started on IV thiamine supplementation 200 mg every eight hours, with follow-up lactate having significantly improved to 5.2 mmol/L (Table 1). Aggressive thiamine replacement was continued for three days. The patient showed improvement in her weakness and had no further neurological or cardiovascular deficits during the course of her hospitalization. She had no further bleeding episodes and was discharged on oral thiamine supplementation.

## Discussion

Lactate is an end-product of anaerobic metabolism, produced by most tissues in the body, and is often used as a prognostic indicator clinically. There are many causes of lactic acidosis, including but not limited to sepsis, hypovolemia, medications, hypoxia, and systemic hypoperfusion. There are three types of lactic acidosis: type A, B, and D. Type A lactic acidosis results from marked hypoxia causing tissue hypoperfusion in the setting of septic shock, cardiopulmonary arrest, or hypovolemia [3]. Clinically, this manifests as impaired mental status, cold, clammy skin, decreased urine output, and hypotension [4]. In the setting of hypoxia, there is insufficient oxygen for aerobic metabolism, forcing pyruvate to be shuttled into anaerobic metabolism, with lactic acid as the byproduct. Type D lactic acidosis is rare and is usually seen in patients with underlying malabsorption, such as short bowel syndrome or small bowel resection. In these cases, bacteria found within the colon metabolize glucose and starches, which otherwise would be absorbed by the small intestine, into D-lactic acid [5]. D-lactic acid, in turn, builds up in the circulation causing metabolic acidosis. Type D lactic acidosis has also been associated with diabetic ketoacidosis [6] and large ingestions of propylene glycol [7]. Type B lactic acidosis, unlike type A, is not associated with hypoxia but rather is due to problems in lactate metabolism resulting in lactate buildup within the circulation. It can be medication-induced, especially with the use of metformin [8], inhaled beta-agonists, and propofol [9]. Type B lactic acidosis is also seen in chronic alcoholism and various hematologic malignancies [10] such as leukemia or lymphoma.

An underdiagnosed and extremely rare cause of type B lactic acidosis is thiamine deficiency. Thiamine, also referred to as vitamin B1, is a water-soluble vitamin primarily found in foods rich in whole grains. The active form of thiamine known as thiamine pyrophosphate (TPP), a phosphorylated ester [11], is an essential cofactor in several biochemical reactions, including the Krebs cycle. Thiamine deficiency can cause neurological impairment, with the three major manifestations being (1) Wernicke’s encephalopathy, which manifests classically as a triad of encephalopathy, gait ataxia, and ophthalmoplegia [12]; (2) dry beriberi, which is characterized by ataxia and peripheral neuropathy; and (3) wet beriberi, which results in lactic acidosis, high output heart failure, and neuropathy [2].

**Figure 1:**
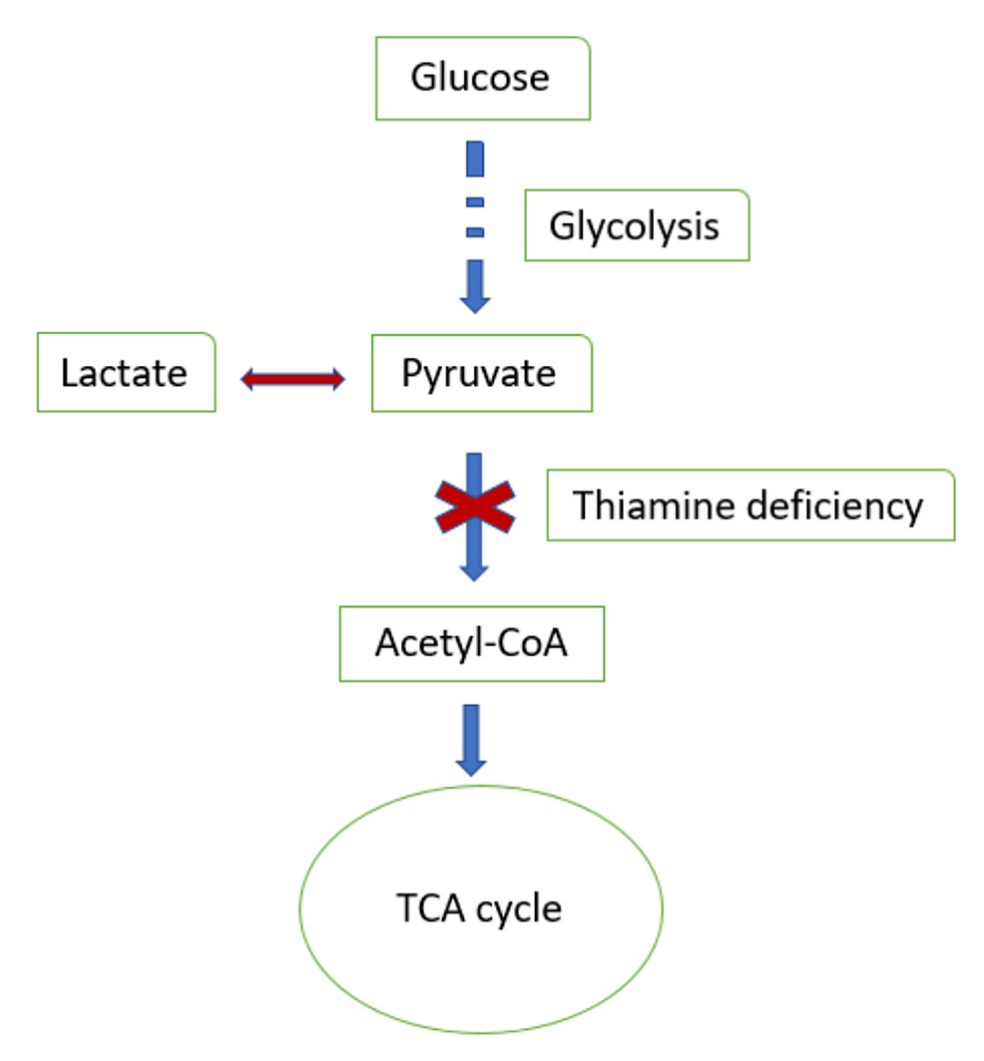
Insufficient thiamine prevents the conversion of pyruvate into acetyl CoA, and rather shunts it towards the production of lactate. CoA: coenzyme A; TCA: tricarboxylic acid

Most cases of thiamine deficiency are seen in critically ill patients who require parenteral nutrition for an underlying condition, alcoholics, or in patients with a history of bowel resection [2]. It is very unusual, especially in the developed world, for it to be seen in patients due to a deficient diet alone, as in our patient. TPP acts as the cofactor for pyruvate dehydrogenase, allowing for the conversion of pyruvate to acetyl coenzyme A (CoA) towards the end of glycolysis. In the absence of TPP, this reaction cannot proceed, and pyruvate gets converted to lactate instead (Figure 1) [11].

As thiamine is essential in carbohydrate metabolism, administering glucose before thiamine can precipitate underlying thiamine deficiency, as seen in our patient.

There are varying guidelines and practices concerning thiamine supplementation in cases of deficiency, and currently, there is no consensus on the dosage or duration of thiamine treatment. In patients with acute thiamine deficiency with cardiovascular or neurologic compromise, IV thiamine 200 mg three times a day until symptom improvement is recommended, after which the patient should be transitioned to 10 mg/day of oral thiamine. In thiamine deficiency with Wernicke-Korsakoff syndrome, IV thiamine 500 mg three times a day is recommended for the first two days, followed by IV thiamine 250 mg daily for three days [13].

## Conclusions

Thiamine deficiency should be considered as a part of the differential diagnosis in patients with refractory lactic acidosis. In such cases, a detailed history should be obtained, especially pertaining to oral intake, particularly in patients from nursing homes. A thorough medical reconciliation is also required as many medications can cause lactic acidosis, as noted above. Clinicians should have a high index of suspicion with a low threshold to supplement thiamine as it is an intervention that is safe, cost-effective, and readily available. Moreover, early intervention can prevent catastrophic outcomes.
